# It Takes Two to Tango: Partnership Between Academia and Long-Term Care

**DOI:** 10.1093/geroni/igaf122.328

**Published:** 2025-12-31

**Authors:** Jan Hamers, Judith Urlings, Hilde Verbeek

**Affiliations:** Maastricht University, Maastricht, Limburg, Netherlands; Living Lab in Ageing and Long-Term Care, Maastricht, Limburg, Netherlands; Maastricht University, Maastricht, Limburg, Netherlands

## Abstract

Older people and their families, health care professionals, policy makers, and educators need to benefit from new advancements and best available evidence to improve their well-being and meet their needs. Researchers aim to have an impact in broader society with their scientific work and influence everyday care practice as well as policy and education. In the Netherlands, we have developed a partnership approach for innovation in long-term care through scientific research which we will present in this symposium. The Limburg Living Lab in Ageing and Long-Term care originated 27 years ago and has proven to be a successful partnership that drives scientific research in long-term care in co-creation with end-users, including older people and their relatives, professional caregivers, managers, policy makers and educators. Thirteen organizations collaborate structurally to improve quality of life, care and work in long-term care. It has three key characteristics, including linking pins (scientific and practice-based), interdisciplinarity and community engamenennt throughout the entire scientific process. Older people and their caregivers together set the agenda for scientific research. This is of great importance to include outcomes that matter to make an impact in practice. Furthermore, we translate results into education via hybrid teaching methods to attract early career professionals and life long learning approaches to support current staff. Using the ecosystem approach, return on investment is large as organizations profit from knowledge created by the network as a whole. Other countries have implemented our model and adapted it to their local context.

